# *catena*-Poly[[di­aqua­bis­(μ_3_-5-carboxyl­ato-1*H*-pyrazole-3-carb­oxy­lic acid-κ^3^ *O* ^3^:*O* ^3^;*O* ^5^)dilithium(I)] monohydrate]

**DOI:** 10.1107/S1600536813026408

**Published:** 2013-10-12

**Authors:** Wojciech Starosta, Janusz Leciejewicz

**Affiliations:** a Institute of Nuclear Chemistry and Technology, ul.Dorodna 16, 03-195 Warszawa, Poland

## Abstract

The basic structural unit of the title polymeric ribbon, {[Li_2_(C_5_H_3_N_2_O_2_)_2_(H_2_O)_2_]·H_2_O}_*n*_, is a centrosymmetric dinuclear complex in which the two Li^I^ ions are bridged by two carboxyl­ato O atoms, to generate a centrosymmetric Li_2_O_2_ core. These are connected into a chain along [01-1] by carboxylic acid–carbonyl-O bonds. The tetra­hedral coordination of the Li^I^ cation is completed by an aqua ligand. The carboxylic acid is involved in an intra-ribbon hydrogen bond. A solvate water molecule showing positional (50:50) disorder is observed. Polymeric ribbons along [01-1] are connected by O—H⋯O, N—H⋯O and O—H⋯N hydrogen bonds into a three-dimensional architecture.

## Related literature

For the structure of the pyrazole-3,5-di­carb­oxy­lic acid hydrate, see: Ching *et al.* (2000 ▶). 
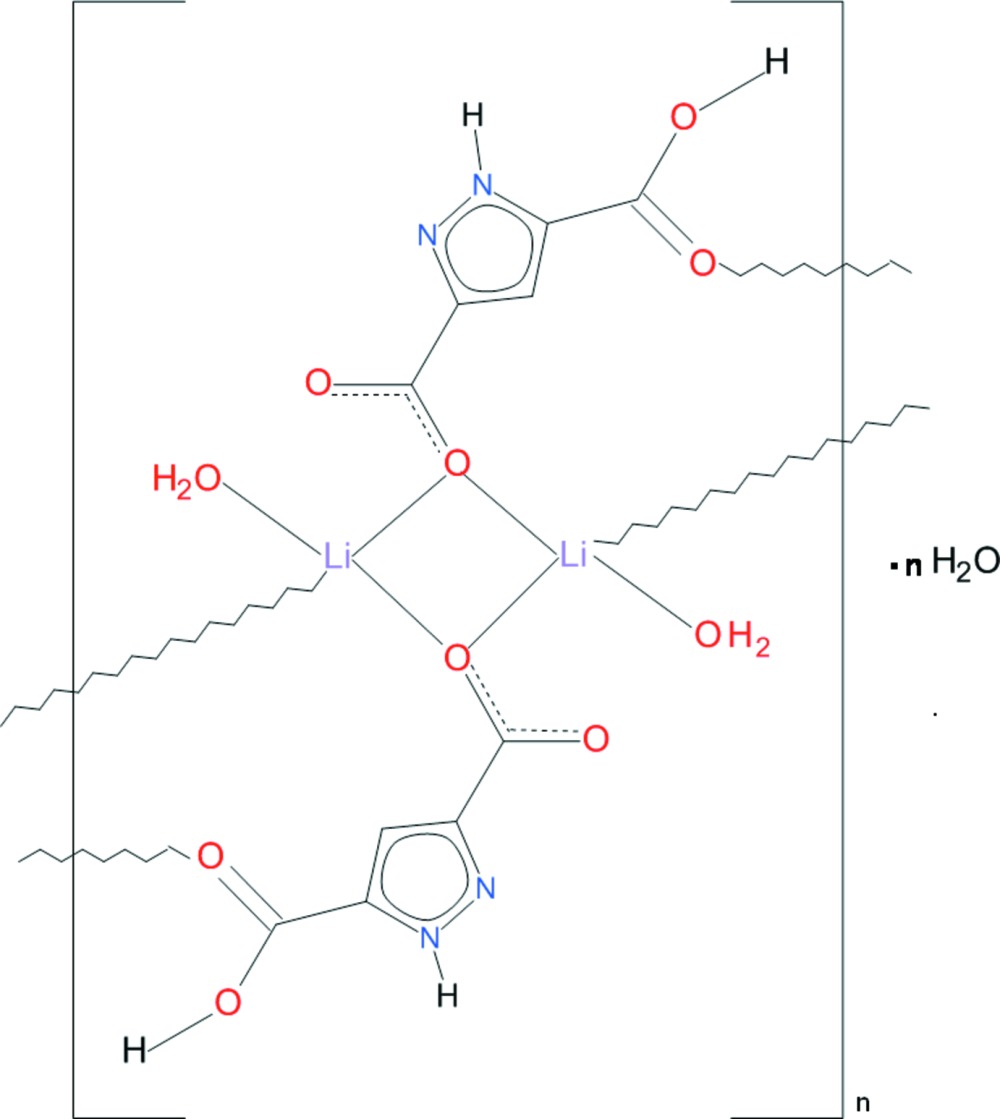


## Experimental

### 

#### Crystal data

[Li_2_(C_5_H_3_N_2_O_2_)_2_(H_2_O)_2_]·H_2_O
*M* *_r_* = 378.12
Triclinic, 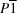

*a* = 7.2610 (15) Å
*b* = 7.5835 (15) Å
*c* = 8.5751 (17) Å
α = 68.38 (3)°
β = 89.07 (3)°
γ = 63.66 (3)°
*V* = 387.19 (13) Å^3^
*Z* = 1
Mo *K*α radiation
μ = 0.15 mm^−1^
*T* = 293 K
0.32 × 0.19 × 0.15 mm

#### Data collection

Kuma KM-4 four-circle diffractometer
Absorption correction: analytical (*CrysAlis RED*; Oxford Diffraction, 2008 ▶) *T*_min_ = 0.963, *T*_max_ = 0.983
2319 measured reflections
2139 independent reflections
1631 reflections with *I* > 2σ(*I*)
*R*_int_ = 0.051
3 standard reflections every 200 reflections intensity decay: 3.2%

#### Refinement

*R*[*F* ^2^ > 2σ(*F* ^2^)] = 0.048
*wR*(*F* ^2^) = 0.139
*S* = 1.04
2139 reflections
148 parameters
4 restraints
H atoms treated by a mixture of independent and constrained refinement
Δρ_max_ = 0.36 e Å^−3^
Δρ_min_ = −0.43 e Å^−3^

### 

Data collection: *KM-4 Software* (Kuma, 1996 ▶); cell refinement: *KM-4 Software*; data reduction: *DATAPROC* (Kuma, 2001 ▶); program(s) used to solve structure: *SHELXS97* (Sheldrick, 2008 ▶); program(s) used to refine structure: *SHELXL97* (Sheldrick, 2008 ▶); molecular graphics: *SHELXTL* (Sheldrick, 2008 ▶); software used to prepare material for publication: *SHELXTL*.

## Supplementary Material

Crystal structure: contains datablock(s) I, New_Global_Publ_Block. DOI: 10.1107/S1600536813026408/kp2459sup1.cif

Structure factors: contains datablock(s) I. DOI: 10.1107/S1600536813026408/kp2459Isup2.hkl

Additional supplementary materials:  crystallographic information; 3D view; checkCIF report

## Figures and Tables

**Table 1:** Selected bond lengths (Å)

Li1—O1	1.948 (3)
Li1—O4^i^	1.910 (3)
Li1—O1^ii^	1.930 (3)
Li1—O5	1.981 (3)

Symmetry codes: (i) 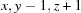; (ii) 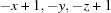.

**Table 2:** Hydrogen-bond geometry (Å, °)

*D*—H⋯*A*	*D*—H	H⋯*A*	*D*⋯*A*	*D*—H⋯*A*
O3—H3⋯O2^iii^	0.82	1.73	2.5159 (16)	160
N1—H1⋯O5^iv^	0.84 (2)	2.02 (2)	2.8233 (17)	161 (2)
O5—H52⋯O6	0.89 (3)	1.94 (3)	2.749 (3)	150 (3)
O5—H52⋯O6^v^	0.89 (3)	2.01 (3)	2.851 (3)	157 (3)
O5—H51⋯N2^vi^	0.93 (3)	1.89 (3)	2.810 (2)	169 (3)
O5—H51⋯O3^vi^	0.93 (3)	2.60 (3)	3.1235 (16)	116 (2)
O6—H62⋯O2^iv^	0.87 (2)	2.03 (3)	2.886 (3)	167 (7)

Symmetry codes: (iii) 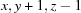; (iv) 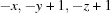; (v) 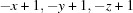; (vi) 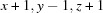.

**Fig. 1.**
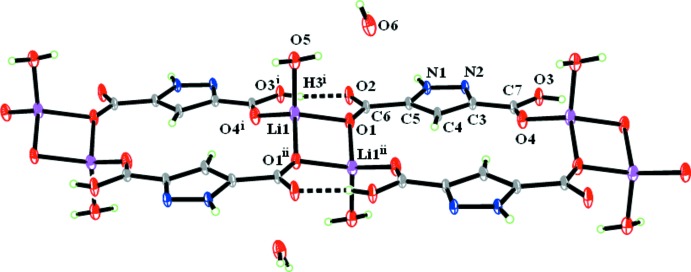
A fragment of a molecular ribbon showing a dinuclear structural building unit of the title complex with atom labelling scheme and 50% probability displacement ellipsoids. Symmetry code: (i) x, y - 1,z + 1; (ii) -x + 1, -y, -z + 1; (iii): -x + 1, -y + 1, -z.

**Fig. 2.**
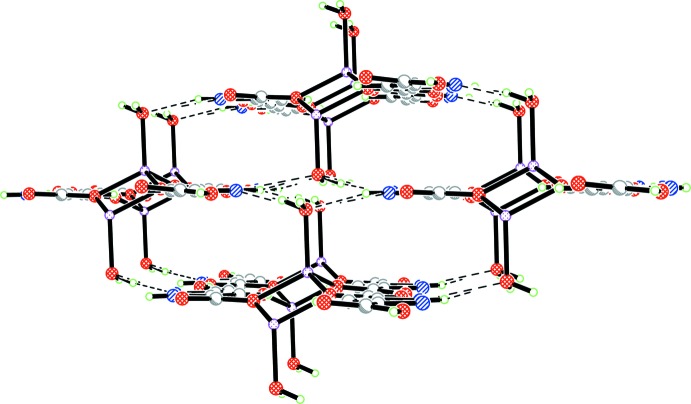
The packing of molecular ribbons via hydrogen bonds viewed along their propagation direction.

## supplementary crystallographic information

### 1. Comment

The structural unit of the title complex is a centrosymmetric dinuclear moiety composed of two Li^I^ ions bridged by two bidentate carboxylato O atoms, each donated by a symmetry related ligand (Fig. 1). The ligand acts in µ_3_ bridging mode since apart from the bidentate O1 atom, the O4 atom of its second carboxylate group is chelated to a Li^(vi)^ ion in the adjacent dimer. In this way a Li^I^ ion is coordinated by the bridging O1 and O1^(ii)^ atoms, the O4^(i)^ from the adjacent dimer and an aqua O5 atom resulting in a distorted tetrahedral geometry. The Li—O bond distances (Table 1) which fall in the range between 1.930 (2) Å and 1.980 (3) Å are typical of Li^I^ complexes with carboxylate and water ligands. The pyrazole ring is planar with r.m.s. of 0.0009 (1) Å; the carboxylate group C6/O1/O2 and C7/O3/O4 make with it dihedral angles of 2.4 (1)° and 5.5 (1)°, respectively. The carboxylate O2 atom is chelating inactive, the O3 remains protonated and participates as a donor in the short hydrogen bond of 2.516 (2) Å to O2^vi^ in an adjacent dimer. Bond distances and bond angles within the pyrazole ring do not differ from those reported in the structure of the parent acid (Ching *et al.*, 2000). The plane of the Li1,O1,Li^(ii)^,O1^(ii)^ dimer core makes a dihedral angle of 36.1° with the ligand plane. The dimeric units linked by carboxylate O4 atoms form molecular ribbons . A solvate water molecule O6 with 50% site occupancy is present in the asymmetric cell resulting in one molecule per a dimer. Moreover, this water molecule shows 0.5/0.5 positional disorder. The ribbons are held together by a system of hydrogen bonds involving coordinated and crystal water molecules the carboxylate/carboxylato groups and pyrazole N ring atoms (Fig. 2, Table 2).

### 2. Experimental

1 mmol of pyrazole-3,5-dicarboxylic acid hydrate and *ca*2 mmol s of lithium hydroxide dissolved in 50 mL of hot, doubly distilled water were boiled under reflux with stirring for six hours and then left to crystallize at room temperature. Colourless single-crystal blocks with two differet shapes deposited after a week. One of the crystals was selected, washed with cold ethanol and dried in the air.

### 3. Refinement

Hydrogen atoms belonging to water molecules, the carboxylate group and hetero-ring N atom were located in a difference map and refined isotropically.

### Crystal data

**Table:**

[Li_2_(C_5_H_3_N_2_O_2_)_2_(H_2_O)_2_]·H_2_O	*Z* = 1
*M**_r_* = 378.12	*F*(000) = 194
Triclinic, *P*1	*D*_x_ = 1.622 Mg m^−^^3^
Hall symbol: -P 1	Mo *K*α radiation, λ = 0.71073 Å
*a* = 7.2610 (15) Å	Cell parameters from 25 reflections
*b* = 7.5835 (15) Å	θ = 6–15°
*c* = 8.5751 (17) Å	µ = 0.15 mm^−^^1^
α = 68.38 (3)°	*T* = 293 K
β = 89.07 (3)°	Blocks, colourless
γ = 63.66 (3)°	0.32 × 0.19 × 0.15 mm
*V* = 387.19 (13) Å^3^	

### Data collection

**Table:**

Kuma KM-4 four-circle diffractometer	1631 reflections with *I* > 2σ(*I*)
Radiation source: fine-focus sealed tube	*R*_int_ = 0.051
Graphite monochromator	θ_max_ = 30.1°, θ_min_ = 2.6°
profile data from ω/2θ scan	*h* = −9→9
Absorption correction: analytical (*CrysAlis RED*; Oxford Diffraction, 2008)	*k* = 0→9
*T*_min_ = 0.963, *T*_max_ = 0.983	*l* = −11→11
2319 measured reflections	3 standard reflections every 200 reflections
2139 independent reflections	intensity decay: 3.2%

### Refinement

**Table:**

Refinement on *F*^2^	Primary atom site location: structure-invariant direct methods
Least-squares matrix: full	Secondary atom site location: difference Fourier map
*R*[*F*^2^ > 2σ(*F*^2^)] = 0.048	Hydrogen site location: inferred from neighbouring sites
*wR*(*F*^2^) = 0.139	H atoms treated by a mixture of independent and constrained refinement
*S* = 1.04	*w* = 1/[σ^2^(*F*_o_^2^) + (0.0979*P*)^2^ + 0.0513*P*] where *P* = (*F*_o_^2^ + 2*F*_c_^2^)/3
2139 reflections	(Δ/σ)_max_ < 0.001
148 parameters	Δρ_max_ = 0.36 e Å^−^^3^
4 restraints	Δρ_min_ = −0.43 e Å^−^^3^

### Special details

**Table:**

Geometry. All e.s.d.'s (except the e.s.d. in the dihedral angle between two l.s. planes) are estimated using the full covariance matrix. The cell e.s.d.'s are taken into account individually in the estimation of e.s.d.'s in distances, angles and torsion angles; correlations between e.s.d.'s in cell parameters are only used when they are defined by crystal symmetry. An approximate (isotropic) treatment of cell e.s.d.'s is used for estimating e.s.d.'s involving l.s. planes.
Refinement. Refinement of *F*^2^ against ALL reflections. The weighted *R*-factor *wR* and goodness of fit *S* are based on *F*^2^, conventional *R*-factors *R* are based on *F*, with *F* set to zero for negative *F*^2^. The threshold expression of *F*^2^ > σ(*F*^2^) is used only for calculating *R*-factors(gt) *etc*. and is not relevant to the choice of reflections for refinement. *R*-factors based on *F*^2^ are statistically about twice as large as those based on *F*, and *R*- factors based on ALL data will be even larger.

### Fractional atomic coordinates and isotropic or equivalent isotropic displacement parameters (Å^2^)

**Table:**

	*x*	*y*	*z*	*U*_iso_*/*U*_eq_	Occ. (<1)
O1	0.31476 (15)	0.19235 (16)	0.41828 (13)	0.0317 (3)	
O3	−0.04302 (15)	1.06450 (16)	−0.29666 (13)	0.0330 (3)	
H3	−0.0066	1.1326	−0.3779	0.050*	
N2	−0.12574 (16)	0.83399 (17)	−0.01238 (13)	0.0229 (2)	
O4	0.30113 (16)	0.84618 (17)	−0.21398 (14)	0.0352 (3)	
O2	−0.02499 (16)	0.33302 (17)	0.43139 (13)	0.0361 (3)	
N1	−0.11472 (16)	0.68180 (16)	0.13403 (13)	0.0219 (2)	
C5	0.08191 (17)	0.51952 (18)	0.19965 (15)	0.0208 (3)	
C4	0.20777 (18)	0.56837 (19)	0.08850 (15)	0.0236 (3)	
H4	0.3512	0.4884	0.0977	0.028*	
C3	0.07157 (18)	0.76495 (18)	−0.04109 (14)	0.0209 (3)	
C7	0.12037 (19)	0.8962 (2)	−0.19328 (15)	0.0230 (3)	
C6	0.12709 (19)	0.33371 (19)	0.36231 (15)	0.0218 (3)	
Li1	0.4462 (4)	−0.0115 (4)	0.6522 (3)	0.0310 (5)	
O5	0.50246 (15)	0.15574 (18)	0.75835 (14)	0.0331 (3)	
O6	0.4576 (4)	0.5520 (4)	0.5472 (4)	0.0493 (6)	0.50
H1	−0.225 (4)	0.700 (4)	0.173 (3)	0.046 (6)*	
H52	0.531 (5)	0.254 (4)	0.683 (4)	0.070 (8)*	
H51	0.630 (5)	0.063 (5)	0.832 (4)	0.078 (9)*	
H62	0.328 (4)	0.602 (6)	0.559 (9)	0.14 (3)*	0.50
H61	0.508 (15)	0.57 (3)	0.624 (19)	0.38 (11)*	0.50

### Atomic displacement parameters (Å^2^)

**Table:**

	*U*^11^	*U*^22^	*U*^33^	*U*^12^	*U*^13^	*U*^23^
O1	0.0179 (4)	0.0260 (5)	0.0233 (4)	0.0000 (4)	0.0017 (3)	0.0056 (4)
O3	0.0216 (5)	0.0280 (5)	0.0247 (5)	−0.0075 (4)	0.0030 (4)	0.0095 (4)
N2	0.0175 (5)	0.0181 (5)	0.0188 (5)	−0.0056 (4)	0.0027 (3)	0.0038 (4)
O4	0.0215 (5)	0.0304 (5)	0.0329 (5)	−0.0091 (4)	0.0097 (4)	0.0044 (4)
O2	0.0212 (5)	0.0292 (5)	0.0296 (5)	−0.0066 (4)	0.0068 (4)	0.0106 (4)
N1	0.0148 (5)	0.0178 (5)	0.0188 (5)	−0.0047 (4)	0.0032 (3)	0.0034 (4)
C5	0.0156 (5)	0.0166 (5)	0.0182 (5)	−0.0050 (4)	0.0025 (4)	0.0019 (4)
C4	0.0150 (5)	0.0183 (5)	0.0218 (5)	−0.0034 (4)	0.0040 (4)	0.0023 (4)
C3	0.0173 (5)	0.0175 (5)	0.0178 (5)	−0.0067 (4)	0.0038 (4)	0.0014 (4)
C7	0.0210 (6)	0.0189 (5)	0.0195 (5)	−0.0082 (4)	0.0047 (4)	0.0006 (4)
C6	0.0172 (5)	0.0173 (5)	0.0177 (5)	−0.0048 (4)	0.0018 (4)	0.0022 (4)
Li1	0.0219 (10)	0.0268 (11)	0.0278 (11)	−0.0074 (9)	0.0077 (8)	0.0006 (9)
O5	0.0173 (4)	0.0320 (5)	0.0309 (5)	−0.0070 (4)	0.0031 (4)	0.0012 (4)
O6	0.0359 (13)	0.0414 (14)	0.0517 (15)	−0.0200 (11)	0.0027 (10)	0.0030 (11)

### Geometric parameters (Å, º)

**Table:**

O1—C6	1.2578 (16)	C5—C6	1.4816 (17)
O1—Li1^i^	1.929 (3)	C4—C3	1.3935 (17)
Li1—O1	1.948 (3)	C4—H4	0.9300
O3—C7	1.2958 (17)	C3—C7	1.4698 (16)
O3—H3	0.8200	Li1—O4^iii^	1.910 (3)
N2—N1	1.3298 (14)	Li1—O1^i^	1.930 (3)
N2—C3	1.3436 (16)	Li1—O5	1.981 (3)
O4—C7	1.2240 (16)	Li1—Li1^i^	2.679 (5)
O4—Li1^ii^	1.910 (3)	O5—H52	0.89 (3)
O2—C6	1.2458 (15)	O5—H51	0.93 (3)
N1—C5	1.3513 (16)	O6—O6^iv^	1.296 (6)
N1—H1	0.84 (2)	O6—H62	0.87 (2)
C5—C4	1.3758 (16)	O6—H61	0.86 (2)
			
C6—O1—Li1^i^	141.01 (13)	O3—C7—C3	113.68 (11)
C6—O1—Li1	128.30 (12)	O2—C6—O1	126.09 (12)
Li1^i^—O1—Li1	87.41 (12)	O2—C6—C5	116.69 (11)
C7—O3—H3	109.5	O1—C6—C5	117.22 (12)
N1—N2—C3	104.63 (10)	O4^iii^—Li1—O1^i^	114.50 (14)
C7—O4—Li1^ii^	136.31 (12)	O4^iii^—Li1—O1	118.27 (14)
N2—N1—C5	112.49 (10)	O1^i^—Li1—O1	92.59 (12)
N2—N1—H1	117.8 (16)	O4^iii^—Li1—O5	111.34 (13)
C5—N1—H1	129.7 (16)	O1^i^—Li1—O5	114.31 (14)
N1—C5—C4	106.99 (10)	O1—Li1—O5	104.38 (13)
N1—C5—C6	120.84 (11)	O4^iii^—Li1—Li1^i^	130.02 (19)
C4—C5—C6	132.17 (11)	O1^i^—Li1—Li1^i^	46.58 (9)
C5—C4—C3	104.29 (10)	O1—Li1—Li1^i^	46.02 (8)
C5—C4—H4	127.9	O5—Li1—Li1^i^	118.50 (16)
C3—C4—H4	127.9	Li1—O5—H52	111.3 (17)
N2—C3—C4	111.59 (11)	Li1—O5—H51	106.9 (16)
N2—C3—C7	120.02 (11)	H52—O5—H51	101 (2)
C4—C3—C7	128.37 (11)	O6^iv^—O6—H62	126 (6)
O4—C7—O3	125.27 (12)	O6^iv^—O6—H61	133 (7)
O4—C7—C3	121.04 (12)	H62—O6—H61	100 (3)

Symmetry codes: (i) −*x*+1, −*y*, −*z*+1; (ii) *x*, *y*+1, *z*−1; (iii) *x*, *y*−1, *z*+1; (iv) −*x*+1, −*y*+1, −*z*+1.

### Hydrogen-bond geometry (Å, º)

**Table:**

*D*—H···*A*	*D*—H	H···*A*	*D*···*A*	*D*—H···*A*
O3—H3···O2^ii^	0.82	1.73	2.5159 (16)	160
N1—H1···O5^v^	0.84 (2)	2.02 (2)	2.8233 (17)	161 (2)
O5—H52···O6	0.89 (3)	1.94 (3)	2.749 (3)	150 (3)
O5—H52···O6^iv^	0.89 (3)	2.01 (3)	2.851 (3)	157 (3)
O5—H51···N2^vi^	0.93 (3)	1.89 (3)	2.810 (2)	169 (3)
O5—H51···O3^vi^	0.93 (3)	2.60 (3)	3.1235 (16)	116 (2)
O6—H62···O2^v^	0.87 (2)	2.03 (3)	2.886 (3)	167 (7)

Symmetry codes: (ii) *x*, *y*+1, *z*−1; (iv) −*x*+1, −*y*+1, −*z*+1; (v) −*x*, −*y*+1, −*z*+1; (vi) *x*+1, *y*−1, *z*+1.
